# Evaluation of Serum for Pathophysiological Effects of Prolonged Low Salinity Water Exposure in Displaced Bottlenose Dolphins (*Tursiops truncatus*)

**DOI:** 10.3389/fvets.2017.00080

**Published:** 2017-06-08

**Authors:** Ruth Y. Ewing, Blair Mase-Guthrie, Wayne McFee, Forrest Townsend, Charles A. Manire, Michael Walsh, Rose Borkowski, Gregory D. Bossart, Adam M. Schaefer

**Affiliations:** ^1^Southeast Fisheries Science Center, National Marine Fisheries Service, National Oceanographic and Atmospheric Administration, Miami, FL, United States; ^2^National Centers for Coastal Ocean Science, National Ocean Service, Charleston, SC, United States; ^3^Bayside Veterinary Hospital, Fort Walton Beach, FL, United States; ^4^Mote Marine Laboratory, Sarasota, FL, United States; ^5^Loggerhead Marinelife Center, Juno, FL, United States; ^6^Aquatic Animal Health, College of Veterinary Medicine, University of Florida, Gainesville, FL, United States; ^7^Department of Biology and Marine Science, Jacksonville University, Jacksonville, FL, United States; ^8^Georgia Aquarium’s Conservation Field Station, St. Augustine, FL, United States; ^9^Georgia Aquarium, Atlanta, GA, United States; ^10^Division of Comparative Pathology, Department of Pathology and Laboratory Medicine, Miller School of Medicine, University of Miami, Miami, FL, United States; ^11^Harbor Branch Oceanographic Institute, Florida Atlantic University, Fort Pierce, FL, United States

**Keywords:** bottlenose dolphin, low salinity, out of habitat/displaced, osmoregulation, pathophysiology

## Abstract

We conducted a retrospective study of serum biochemistry and hematologic findings from displaced, out-of-habitat bottlenose dolphins (*Tursiops truncatus*) exposed to various low salinity environments in waters along the southern United States including southeastern Atlantic and northern Gulf of Mexico. Serum sodium, chloride, and calculated osmolality were significantly lower and below reference ranges in displaced animals compared to free-ranging case control animals. This suggests clinical hyponatremia, hypochloremia, and hypo-osmolality due to an uptake of low saline water from the environment. In addition, significant differences were found in other serum chemistry variables, although none were outside of normal reference ranges for non-controlled free-ranging animals. Multiple linear regressions demonstrated the degree of salinity had a greater pathophysiologic response than the duration of fresh water exposure. The Na/Cl ratio and bicarbonate were the only variables that were significantly modulated by exposure duration. These findings suggest that the degree of salinity is a critical factor when assessing and managing care for dolphins chronically exposed to low salinity water. Results from this study indicate that changes in various biochemical parameters can be used to determine fresh water exposure and aid in determining the treatment for animals recovered from low salinity waters.

## Introduction

Common coastal bottlenose dolphins (*Tursiops truncatus*) are osmoregulators capable of regulating and modulating their internal water and electrolyte concentrations to maintain an optimal osmotic pressure to sustain cellular function in hyperosmotic environments. This adaptation is elegantly exhibited in these animals specifically in a hypersaline environment such that evolutionary adaptations are geared toward the conservation (or net gain) of freshwater, thereby avoiding dehydration while excreting excess electrolytes. Coastal bottlenose dolphins generally inhabit North American coastal lagoon types (e.g., ocean continental shelf to brackish estuaries including near river estuaries, coastal lagoons, intracoastal water ways, and bayous) with salinities that range from 18 to 35 ppt, in water <25 m deep (1, 2).

Marine mammals are defined as “out-of-habitat” (OOH) when they are found out of their normal distributional and/or habitat range and thereby would likely perish due to environmental factors and/or resource limitations (3). Coastal bottlenose dolphins are known to occasionally venture into riverine systems either by short migration (“limited invasion”), happenstance, or by weather system displacement via storm surge (3, 4). In the event that an animal is classified as OOH, unable to return to areas of higher salinity, and/or is suffering from the ill effects of fresh water exposure, the National Marine Fisheries Service (NMFS), in conjunction with partner institutions, may initiate rescue response efforts if logistically feasible. During these rescue-capture efforts, animals are given a physical examination and blood is collected for hematologic and serum chemistry analysis to determine if the dolphin is a candidate for rehabilitation, euthanasia, or release into a more suitable habitat.

To date, the confirmation of fresh water intoxication has relied upon visualization of skin lesions consistent with direct degenerative change (hydropic degeneration), peeling, dolphin poxvirus recrudescence, or secondary infections from opportunistic pathogens; however, these changes can be obscured by post-mortem changes (4–7). We hypothesize that measurement of biomarkers in serum, including minerals, electrolytes, urea nitrogen, and creatinine, should yield evidence of fluid derangements associated with exposure to decreased salinity water (8). To address this hypothesis, serum biochemistry and hematologic findings of *Tursiops* found OOH were compared to findings from a matched cohort of healthy, free-ranging *Tursiops*.

## Methods

### Study Site and Animals

Dolphins defined as OOH between 2001 and 2016 were identified for inclusion in the study from the NMFS Marine Mammal Health and Response Program National Database. From those, data were analyzed from 14 individuals that were exposed to salinities below 11 ppt for >5 days. Animals were rescued by a coalition of Southeast Marine Mammal Stranding Network Members from waterways along the U.S. southeastern Atlantic coast and northern Gulf of Mexico and evaluated for hematologic and serum biochemistry parameters (Figures S1 and S2 in Supplementary Material). Duration of known low salinity exposure was defined as the number of days that elapsed between when NMFS was first notified of the OOH animal until its capture date. Twelve animals were found within a riverine system of low salinity to freshwater (0 ppt). The remaining two animals were displaced by tropical storm/hurricane into an impoundment and dredge canal, respectively. Various methods were used to measure the salinity at the sites where the animals were observed. Salinity was measured using a hand held YSI (YSI Inc., Yellow Springs, OH, USA) probe at approximately mid water column based on location depth and reported in ppt upon initial capture for eight displaced and all control individuals. Additional salinity measures for three OOH animals were taken at the surface using a refractometer. At the remaining case sites salinity was not measured; however, reduced salinity was presumed based on inland site location and/or the presence of skin changes consistent with freshwater exposure (7). The OOH case animals were captured by shallow (3ʺ–5ʺ) or deep water (>5ʺ) sets as the site location logistically dictated typically using a seine net, 400ʺ in length ×18ʺ in depth with a float-line at the surface and double lead line on the bottom, methods previously described (9). The animals were then transferred to a boat for physical examination and routine phlebotomy (described below).

The control group (matched on gender and length) was selected from data previously collected during the bottlenose dolphin Health and Environmental Risk Assessment (HERA) from the Indian River Lagoon, FL and Charleston, SC between 2003 and 2015 using previously established protocols and techniques (10). All HERA capture and sample collections were approved under the National Marine Fisheries Service of Scientific Research Permit No. 998-1678 and 14352. Additionally, the Florida Atlantic University Institutional Animal Care and Use Committee approved animal handling protocols. Only individuals who were classified as healthy by a panel of marine mammal veterinarians (11), based on physical exam, serum chemistry, hematology, and cytology data were included as controls.

### Blood Chemistry and Hematology

The methods for blood collection and hematologic and serum chemistry assessment followed those described previously (9, 10). Briefly, blood samples were drawn from the periarterial venous rete in the flukes using a 19-gage needle and placed into 10 ml serum separator and EDTA anticoagulant vacutainer tubes (Becton, Dickinson and Company). Samples for serum were allowed to clot, centrifuged for 15 min at 1,233 × *g*, and then serum aliquots were placed in cryovials (Corning Inc., Life Sciences, Lowell, MA, USA) for later analysis. Hematologic parameters were assessed on automated hematology analyzers (i.e., Bayer ADVIA 120, Bayer Diagnostics) and included white blood cell counts (WBC), red blood cell counts (RBC), hemoglobin, hematocrit, mean corpuscular volume (MCV), mean corpuscular hemoglobin (MCH), MCH concentration (MCHC), and platelet counts. Serum biochemistry and electrolyte variables were run using automated chemistry analyzers (i.e., Hitachi 917, Roche and Roche Diagnostics C501 chemistry analyzer) and included glucose, blood urea nitrogen (BUN), creatinine, carbon dioxide, uric acid, triglycerides, cholesterol, total bilirubin, alkaline phosphatase (AP), lactase dehydrogenase (LDH), aspartate transaminase (AST), alanine transaminase (ALT), total protein, albumin, globulin, iron, total iron binding capacity, iron saturation, creatine kinase (CK), gamma glutamyltransferase (GGT), amylase, lipase, sodium (Na), potassium (K), chloride (Cl), calcium, phosphorus, bicarbonate (HCO_3_), and magnesium. In addition, the calculated variables from serum parameters were calculated using the reported blood values according to the Worthley equation (12, 13) for osmolality and a reference equation for the anion gap (14):

$$\text{Osmolality}=(2[{\text{Na}}^{+}(\text{mEq}/\text{L})])+([\text{Glucose (mg/dl)}]/18)+([\text{BUN (mg}/\text{dl)}]/2.8).$$

$$\text{Anion Gap}=([{\text{Na}}^{+}+{\text{K}}^{+}])-([{\text{Cl}}^{-}+{\text{HCO}}_{3}^{-}]).$$

The blood samples of the OOH animals were analyzed by a variety of clinical laboratories at the discretion of the responding veterinarian and/or depending on event location. For hematology and serum biochemistry analysis, these laboratories included: Animal Health Diagnostic Center, Veterinary College, Cornell University; North Carolina State College of Veterinary Medicine Clinical Pathology Lab; University of Florida Veterinary Medical Teaching Hospital; Ft. Walton Beach Medical Center Clinical Laboratory; Sarasota Memorial Hospital; Clinical Pathology Laboratory at Memorial Hospital Gulfport; and Baptist Medical Center (Jacksonville, FL, USA). The blood samples from the control animals were all analyzed at Cornell University, College of Veterinary Medicine Animal Health Diagnostic Center.

### Statistics

Descriptive statistics (mean and SDs) were calculated for all blood chemistry and hematology measures from the low salinity exposure group for comparison with control animals. Inter-lab variability was examined among the exposed individuals using an analysis of covariance to compare mean blood values across laboratories while controlling for gender and exposure duration. All data were assessed for normality prior to utilizing an independent sample *t*-test to compare values between low salinity exposed individuals and healthy controls. Multiple linear regressions were applied to examine relationships between the measured salinity and duration of exposure to low salinity on all hematologic and serum chemistry parameters. The analysis enabled an assessment of potential effects of low salinity and/or duration of low salinity exposure on the pathophysiological response among exposed individuals who had complete records for both salinity and blood measures. Finally, a multivariate linear regression with a stepwise addition was used to include exposure time, salinity, and an interaction term for each blood parameter on exposed individuals. Results were considered statistically significant at *p* < 0.05. All analyses were completed using SPSS version 23.0 (IBM Corp., Armonk, NY, USA).

## Results

Fourteen dolphins that had been exposed to low salinity environments for >5 days were compared with 14 matched, healthy, free-ranging controls. Matching eliminated differences in length and gender between groups with mean salinity as the only significantly different (*p* < 0.01) variable between exposed (2.7 ± 3.5 ppt) and normal controls (28.7 ± 7.0 ppt; Table 1).

**Table 1:** **Comparison mean (±SD) of bottlenose dolphins with prolonged exposure (>5 days) to low salinity water (salinity <10 ppt) and length and sex matched, healthy, free-ranging control dolphins from Health and Environmental Risk Assessment (HERA) study in Indian River Lagoon, FL and Charleston, SC**.

	HERA controls (*n* = 14)	Exposed (*n* = 14)	*p*-Value
Gender male	9 (64.3%)	9 (64.3%)	n/a
Gender female	5 (35.7%)	5 (35.7%)	n/a
Length (cm)	212.31 (31.85)	217.23 (30.94)	0.72
Salinity (ppt)*	28.68 (6.97)	2.70 (3.50)	<0.01
Approximate exposure (days)		22.20 (14.89)	n/a

*Denotes significant difference (*p* < 0.05) between HERA controls and exposed individuals.

Glucose, HCO_3_, total bilirubin, ALT, total protein, and globulin levels were significantly higher among low salinity exposed individuals compared to normal, healthy controls (Table 2). In contrast, osmolality and AP were significantly lower among OOH individuals. No significant differences were observed in hematology values between OOH and control animals (Table 3). Electrolyte changes included significantly lower Na, Cl, and Na/K ratio in low salinity exposed individuals, while Na/Cl ratio was significantly higher when compared to the control population (Table 4).

**Table 2:** **Comparison of serum chemistry parameters mean (±SD) of bottlenose dolphins with prolonged exposure (>5 days) to low salinity water (salinity <10 ppt) and length and sex matched, healthy, free-ranging control dolphins from Health and Environmental Risk Assessment (HERA) study in Indian River Lagoon, FL and Charleston, SC**.

		HERA controls (*n* = 14)		Exposed (*n* = 14)		
Blood chemistry	Units	Mean	SD	Mean	SD	*p*-Value
Glucose*	mg/dL	96.31	16.09	133.79	40.14	0.01
Carbon dioxide	mEq/L	23.67	7.57	25.00	8.46	0.83
Bicarbonate*	mEq/L	22.80	3.82	28.00	1.00	<0.01
BUN	mg/dL	56.50	10.94	68.23	37.90	0.27
Creatinine	mg/dL	1.12	0.21	1.29	0.52	0.42
Calc osmolality*	cOsm/kg	319.88	18.74	294.21	19.50	<0.01
Uric acid	mg/dL	0.32	0.30	0.90	0.77	0.16
Cholesterol	mg/dL	165.71	22.87	173.80	62.65	0.75
Triglycerides	mg/dL	87.00	34.31	153.00	121.41	0.28
Bilirubin, total*	mg/dL	0.09	0.03	0.18	0.14	0.04
AP*	U/L	357.92	265.87	163.07	101.05	0.02
Lactase dehydrogenase	U/L	880.00	362.05	1124.50	965.61	0.61
Aspartate transaminase*	U/L	205.00	54.51	325.42	151.04	0.02
Alanine transaminase*	U/L	40.38	17.26	77.93	60.95	0.05
Total protein*	g/dL	7.22	0.68	8.06	0.85	0.01
Albumin	g/dL	4.23	0.77	3.77	1.15	0.21
Globulin*	g/dL	2.97	0.76	4.08	1.32	0.01
A/G ratio		1.60	0.78	1.21	0.61	0.17
Anion gap	mEq/L	23.00	6.27	26.25	5.13	0.17
Iron	µg/dL	109.80	31.52	128.63	44.04	0.21
TIBC	µg/dL	251.00	57.46	378.00	126.76	0.22
Iron saturation	%	44.50	10.87	50.33	21.46	0.42
CK	U/L	181.50	70.88	308.77	251.77	0.08
GGT	U/L	26.83	9.43	32.89	12.47	0.22
Amylase	U/L	8.50	7.14	3.75	0.96	0.17
Lipase	U/L	25.33	29.50	7.50	6.40	0.16

*Denotes significant difference (*p* < 0.05) between HERA controls and exposed individuals.

**Table 3:** **Comparison of hematologic parameters mean (±SD) of bottlenose dolphins with prolonged exposure (>5 days) to low salinity water (salinity <10 ppt) and length and sex matched, healthy, free-ranging control dolphins from HERA study in Indian River Lagoon, FL and Charleston, SC**.

		HERA controls (*n* = 14)		Exposed (*n* = 14)		
Hematology	Units	Mean	SD	Mean	SD	*p*-Value
White blood cell counts	×10^3^/µL	9.94	2.62	11.61	7.57	0.54
Red blood cell counts	×10^3^/µL	3.67	0.13	3.55	0.62	0.58
Hemoglobin	g/dL	13.92	0.86	13.33	2.47	0.55
Hematocrit	%	42.08	2.53	40.87	7.36	0.69
Mean corpuscular volume	fL	114.31	6.80	116.25	7.91	0.43
Mean corpuscular hemoglobin (MCH)	pg	39.23	1.74	38.46	2.02	0.38
MCH concentration	g/dL	34.23	0.93	33.46	1.67	0.20
Platelet count	×10^3^/µL	196.92	65.15	208.38	77.86	0.64

**Table 4:** **Comparison of electrolyte variables mean (±SD) of bottlenose dolphins with prolonged exposure (>5 days) to low salinity water (salinity <10 ppt) and length and sex matched, healthy, free-ranging control dolphins from Health and Environmental Risk Assessment (HERA) study in Indian River Lagoon, FL and Charleston, SC**.

		HERA controls (*n* = 14)		Exposed (*n* = 14)		
Electrolytes	Units	Mean	SD	Mean	SD	*p*-Value
Sodium*	mEq/L	156.31	9.05	142.57	9.60	<0.01
Potassium	mEq/L	4.08	0.44	4.40	1.24	0.34
Na/K*	ratio	38.58	3.74	33.99	6.73	0.03
Chloride*	mEq/L	114.77	6.64	97.29	11.87	<0.01
Na/Cl*	ratio	1.36	0.04	1.48	0.10	<0.01
Calcium	mg/dL	9.25	0.56	9.19	1.01	0.94
Phosphorus	mg/dL	5.15	0.73	6.66	3.47	0.18
Magnesium	mg/dL	1.50	0.16	1.50	0.26	1.00

*Denotes significant difference (*p* < 0.05) between HERA controls and exposed individuals.

Multiple linear regressions were developed in order to examine the relationship between the measured salinity, duration of low salinity exposure, and all blood concentrations in order to establish potential effects of dose and duration on the pathophysiological response among exposed individuals. When adjusting for salinity and duration (days) of low salinity exposure, salinity concentration was a significant linear driver of the AST change with a magnitude of a 25.6 U/L increase per 1 ppt decrease in salinity (Table 5). Amylase was also impacted by salinity only with a decrease of 2.18 U/L with every 1 ppt decrease in salinity. In contrast, the Na/Cl ratio was significantly associated with duration of exposure with a sample increase, 0.01 with each additional day of exposure. Bicarbonate also increased with increasing duration of exposure by 1.29 mEq/L per day of exposure. Finally, MCH was affected by both low salinity and duration of exposure such that for every 1 ppt decrease in salinity, there was a 0.59 pg decrease in MCH. However, for each additional day of exposure, the MCH value also increased by 0.15 pg; therefore, the net effect of each 1 ppt decrease in salinity was a 0.41 pg decrease in MCH.

**Table 5:** **Linear relationship between clinical indicators of health, duration of exposure, and salinity among displaced bottlenose dolphin using stepwise addition multivariate linear regression**.

	Salinity		Duration		
Dependent variable	β	*p*-Value	β	*p*-Value	*R*^2^
**Blood chemistry**					
Glucose	−12.56	0.16	1.36	0.30	0.26
Blood urea nitrogen	−6.97	0.26	0.43	0.65	0.21
Bicarbonate*	−4.43	0.68	1.29	<0.01	1.00
Creatinine	−0.03	0.83	−0.01	0.90	0.02
Calculated osmolality	3.85	0.38	0.19	0.77	0.23
Cholesterol	12.18	0.28	0.16	0.92	0.35
Bilirubin, total	−0.02	0.55	−0.01	0.59	0.20
Alkaline phosphatase	16.54	0.31	−3.87	0.14	0.29
Lactase dehydrogenase	16.28	0.91	17.67	0.44	0.88
Aspartate transaminase*	−25.61	0.02	1.50	0.27	0.68
Alanine transaminase	0.59	0.95	−0.01	0.99	0.01
Total protein	0.04	0.83	−0.01	0.62	0.04
Albumin	0.19	0.48	−0.05	0.21	0.21
Globulin	−0.15	0.58	0.04	0.38	0.13
A/G ratio	0.13	0.28	−0.03	0.15	0.32
Anion gap	−1.44	0.47	0.24	0.53	0.09
Iron	−11.23	0.46	5.56	0.25	0.75
Creatine kinase	−30.13	0.10	5.76	0.06	0.51
Gamma glutamyltransferase	2.00	0.07	−0.30	0.08	0.80
Amylase*	2.18	0.04	−0.03	0.09	0.99
**Hematology**					
White blood cell counts	−1.01	0.64	−0.21	0.57	0.26
Red blood cell counts	0.10	0.38	0.01	0.59	0.42
Hemoglobin	0.59	0.42	−0.03	0.81	0.33
Hematocrit	1.16	0.42	−0.05	0.82	0.22
Mean corpuscular volume	0.35	0.86	−0.31	0.36	0.29
Mean corpuscular hemoglobin* (MCH)	0.59	0.04	−0.15	0.01	0.91
MCH concentration	0.62	0.12	0.01	0.80	0.79
Platelet count	5.83	0.51	3.31	0.19	0.92
**Electrolytes**					
Sodium	1.87	0.39	0.11	0.73	0.24
Potassium	0.08	0.45	−0.01	0.38	0.12
Na/K	−0.33	0.73	0.14	0.34	0.14
Chloride	0.07	0.97	0.57	0.11	0.26
Na/Cl*	0.01	0.30	−0.01	0.01	0.65
Calcium	0.12	0.09	−0.02	0.07	0.42
Phosphorus	0.07	0.68	−0.01	0.67	0.05

*Denotes significant difference (*p* < 0.05).

## Discussion

Recent severe weather, climatic, and human-induced disturbances have resulted in bottlenose dolphins being displaced from their normal habitat (i.e., in marine waters with salinity of >18 ppt) (3, 6, 7). The extended habitation in waters of significantly lower salinity presents potential health challenges to dolphins, including pathophysiologic changes that could result in mortality. This study demonstrated that serum sodium, chloride, and calculated osmolality were significantly lower in OOH animals relative to healthy, free ranging, control animals, indicating hypo-osmolality likely due to uptake of low saline water. Ridgway and Venn-Watson (15) demonstrated similar findings in *Tursiops* gavaged with 2–4 L of fresh (deionized) water, such that plasma sodium, chloride, and osmolality decreased below reference ranges and remained decreased to their last sampling at 24 h post ingestion. Similar findings are reported for manatees and pinnipeds as reviewed in Suzuki and Ortiz (16). In general, marine mammal freshwater loading results in diuresis with associated hyponatremia, hypochloremia, and hemodilution because these animals cannot excrete a hypo-osmolar urine to that of plasma, suggesting marine mammals do not have mechanisms to efficiently conserve electrolytes while excreting excess water (16). Fatal water intoxication is recognized as acute intoxication resulting from rapid excessive water intake which in humans has been associated with overhydration subsequent to physical exertion or heat stress, psychogenic polydipsia, child abuse, and iatrogenic gastric lavage as noted by Farrell and Bower (17) and documented experimentally in harbor seals by Ladd and colleagues (18). There are, however, no other hematologic or clinical chemistry data on chronic fresh water exposure in marine cetaceans.

In terrestrial species, hormones (including aldosterone and cortisol) mediate longer-term water regulation and homeostasis. These hormones were not consistently evaluated in the animals utilized in our analyses. However, the few animals for which these data were available had high aldosterone (1,265 and 287 vs 101 µg/dL in free-ranging control animals) and cortisol (4.3 vs 2.0 µg/dL in free-ranging control animals) levels consistent with predictions (16). Interestingly, the significant elevation in HCO_3_ suggests potential metabolic alkalosis with an elevated anion gap compared to the control group. These elevations could be attributed to concomitant hypochloremia (in most cases) induced renal HCO_3_ retention and/or elevated aldosterone mediated retention. Onsite blood gas analysis coupled with lactate levels could improve assessment of the serum HCO_3_ and anion gap to evaluate acid–base status. Further investigation into the hormonal and compensatory acid–base mechanisms is needed to better understand the homeostatic and stress response to prolonged exposure to fresh water/reduced salinity environments in cetaceans.

Significant differences were noted in various other serum chemistry values (e.g., glucose, bilirubin, ALT, AST, total protein, and globulins were greater than HERA controls); however, none of these differences fell out of the normal reference ranges (19). Based on body length, eight of the 14 OOH animals were classified as juveniles or calves. The expected level of AP in these animals would be higher due to rapid bone growth and remodeling, which would have been reflected in the average AP for OOH animals. AP was significantly lower in OOH dolphins than free-ranging controls and likely represented biologically significant decrease in this enzyme activity. The lower AP suggests that growth may have been slowed significantly perhaps due to competing metabolic demands to maintain homeostasis while OOH. Additionally, lower AP could be related to a negative energy balance attributed to inanition (reduced familiar prey availability) or indicative of a concomitant endotoxemia or diseased state (20–22).

Caldwell and Caldwell (4) describe instances of dolphins making excursions about 75 miles up the St. Johns River into fresh water (near the city of Palatka, FL, USA) theorizing that the higher calcium chloride concentrations possibly aided in physiologic compensation for the decreasing salinity thereby increasing osmoregulatory tolerance. However, intercedent rescues and evaluations in the early 1970s were not common limiting any comparison beyond the observational. Data from sea otters and pinnipeds suggest protein catabolic end products (urea and/or ammonia) contribute to solute homeostasis (16). Future study is needed to understand the role of metabolic compensatory mechanisms in water homeostasis during periods when animals are in a low salinity habitat. Finally, the relationships between exposure time, salinity changes, and disease need further investigation to help inform management decisions regarding response, rescue, and rehabilitation practices.

Data from the current study support the use of electrolytes and serum chemistries as biomarkers for documenting prolonged (>5 days) exposure to low salinity environments and provide confirmation of measurable physiologic changes in dolphins associated with being in a reduced salinity habitat. In addition, the results of the multiple linear regressions demonstrate that in most cases a reduced salinity measurement, particularly a low value nearing that of a truly freshwater environment (0 ppt), had a greater impact on the individuals’ pathophysiological response to low salinity exposure than the actual duration of exposure and therefore is a key factor in therapeutic management. Only the Na/Cl ratio was significantly impacted by duration; however, the magnitude of this change was marginal (β = 0.01). Collection of a complete set of blood, as well as urine, parameters from more animals found OOH could help elucidate these relationships and perhaps help define how long animals have been OOH.

The retrospective nature of this study and the varying OOH conditions to which the individual dolphins were subjected present potential limitations. While the methodology for analysis and species specific reference ranges for clinical parameters are standardized, differences in sample processing were impossible to control. While not precluding comparison of data analyzed from various laboratories, the potential for confounds and bias due to the different instrumentation, especially for particular variables in the blood, must be considered (19). The reduced number of individuals fitting the criteria for inclusion in this study prevented a more detailed breakdown of biochemical changes in relation to time exposed to fresh water. These data also do not control for pre-existing, concurrent, or subclinical disease in these animals. Many of the OOH animals had integument lesions, consistent with prolonged fresh water exposure some with secondary pathogen infections. However, necropsy data are only available for two animals as the rest were either rehabilitated and released or released shortly after capture and relocation. The quantitative measures of salinity for exposed individuals were made at a single point in time and therefore, may not have captured potential changes in the salt content that could result from tidal changes, rainfall runoff nor presence of saltwater wedges or stratification in the water column. Also variations in prey availability to the dolphins may have influenced their electrolyte levels. Finally, the precise exposure duration to a low salinity environment is not known for each animal prior to its being reported to NMFS. Despite these limitations, the results from the current study represent the first attempt to identify hematologic and serum biochemistry effects of prolonged fresh water exposure on wild dolphins where controlled fresh water exposure experiments are not possible or ethical. These data support our hypothesis that measurement of electrolytes is useful for documenting exposure of dolphins to low salinity conditions. Moreover, dolphins experience a measurable physiologic change associated with being in a reduced salinity habitat. Additionally, data suggest a potential dose response relationship between salinity and the magnitude of change among electrolyte endpoints in displaced dolphins. The variations in biochemical parameters can now be used as biomarkers of prolonged fresh water exposure that provides significant additional information for managers and veterinarians making decisions regarding treatments, relocations, etc. of stranded and/or OOH dolphins.

## Ethics Statement

This study was carried out on biological samples, collected from free-ranging bottlenose dolphins, conducted under NMFS, Scientific Research Permits for both HERA control and exposed dolphins. HERA capture and sample collections were approved under the NMFS of Scientific Research Permit No. 998-1678 and 14352. Additionally, the Florida Atlantic University Institutional Animal Care and Use Committee approved animal handling protocols for the HERA baseline health studies. The NMFS exposed animal samples were collected under various Scientific Research and Enhancement NMFS Permits spanning multiple years 2001–2003: 932-1489-01/PRT009526, 2004: 932-1489-05, 2005–2007: 932-1489-08, 2008: 932-1489-09, 2009: 932-1489-10, 2010: 932-1905/MA-009526, 2014: 932-1905-01/MA-009526-1, 2016: 18786.

## Author Contributions

All contributing authors have approved this work for publication. Individually contributions were distributed as follows: study concept—RE, BM-G; study design—RE, BM-G, WM, GB, AS; sample data acquisition and interpretation—RE, FT, CM, MW, RB, GB; statistical analysis and interpretation—AS; manuscript preparation—RE, AS; manuscript review—BM-G, WM, FT, CM, MW, RB, GB.

## Conflict of Interest Statement

The authors declare that the research was conducted in the absence of any commercial or financial relationships that could be construed as a potential conflict of interest.

## Abbreviations

ANCOVA, analysis of covariance; EDTA, ethylenediamine tetraacetic acid; HERA, Health and Environmental Risk Assessment; NMFS, National Marine Fisheries Service; OOH, out of habitat; ppt, parts per thousand; SPSS, Statistical Package for the Social Sciences; YSI, Yellow Springs Instrument Company (YSI Inc.).
