# Supplementary material for: Evaluation of Serum for Pathophysiological Effects of Prolonged Low Salinity Water Exposure in Displaced Bottlenose Dolphins (Tursiops truncatus)
Source: Front Vet Sci. 2017 Jun 8;4:80. doi: 10.3389/fvets.2017.00080 (PMC5462898; doi:10.3389/fvets.2017.00080)
Supplement: Supplementary file 1 [file Data_Sheet_1.DOCX]

***Supplemental material***

**Evaluation of Serum for Pathophysiological Effects of Prolonged Low Salinity Water Exposure in Displaced Bottlenose Dolphins (*Tursiops truncatus*)**

***Ruth Y. Ewing^1^, Blair Mase-Guthrie^1^, Wayne McFee^2^, Forrest Townsend^3^, Charles A. Manire^4^, Michael Walsh^5^, Rose Borkowski^6^, Gregory D. Bossart ^7^ and Adam M. Schaefer^8^**

*Corresponding Author: ruth.ewing@noaa.gov

Supplementary Figure 1. Locations of displaced dolphins along the southeastern Atlantic coast and western Florida.


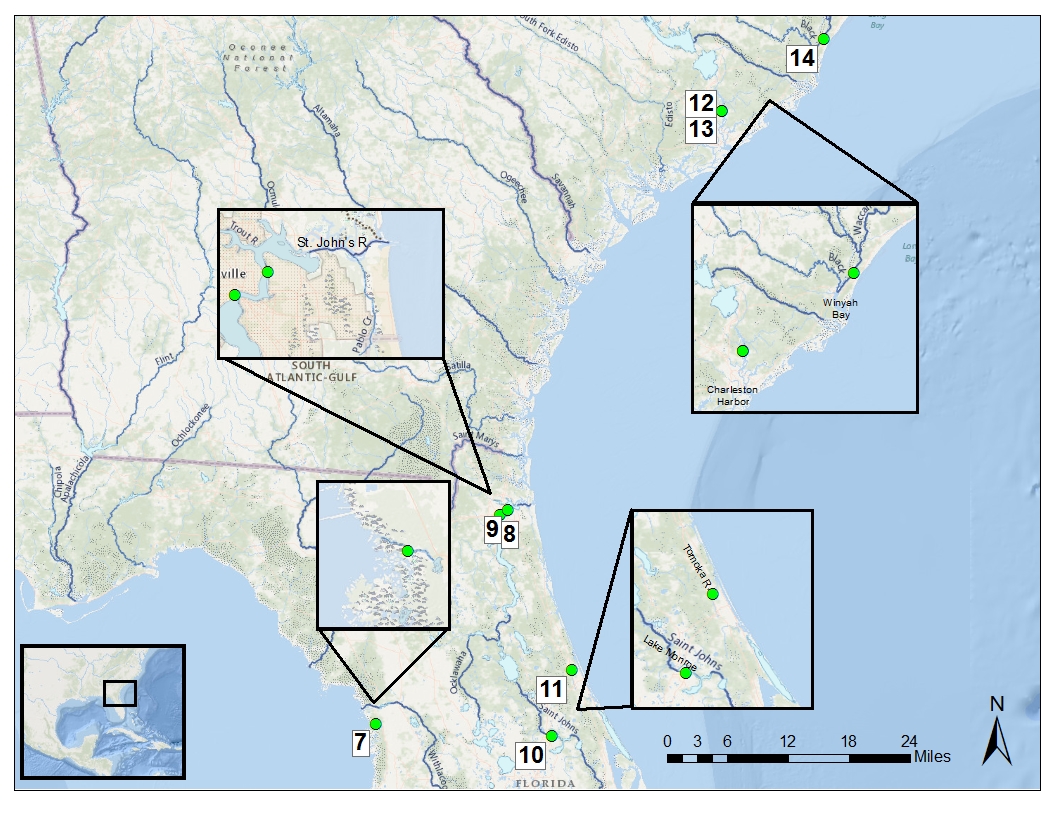


Supplementary Figure 2. Locations of displaced dolphins along the Gulf of Mexico coast from the Florida panhandle to Texas.


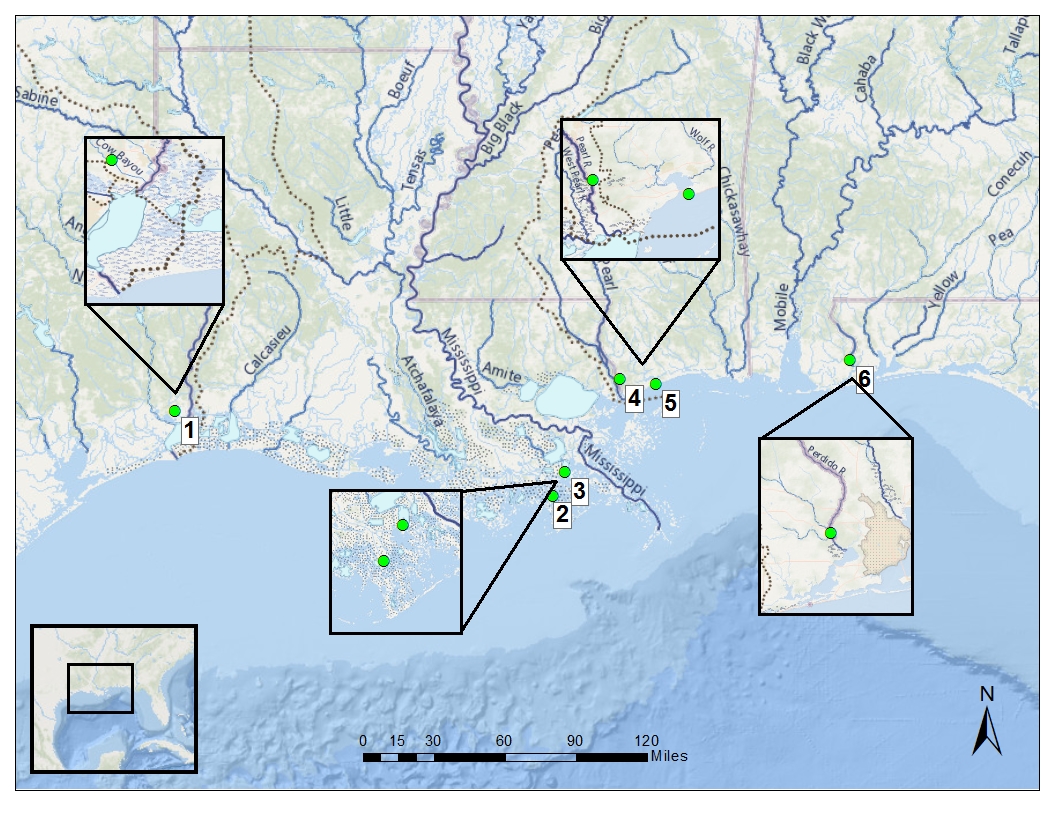


| Map Key | ID | River name | Salinity |
| --- | --- | --- | --- |
| 1 | SER14-00694 | Cow Bayou | 1ppt |
| 2 | SER03-661 | Bayou Lafourche | 5ppt |
| 3 | SER07-338 | Bayou Perot | unk |
| 4 | SER05-747 | Pearl River (dredge canal) | 1ppt |
| 5 | SER16-00019 | Henderson Point | 3.5-5ppt |
| 6 | SER16-00162 | Perdido River | 6.2ppt |
| 7 | SER03-180 | Salt River | 0ppt |
| 8 | SER07-172 | St. Johns River | 11ppt |
| 9 | SER08-0590 | St. Johns River | 1-4ppt |
| 10 | SER09-0022 | Lake Monroe, South shore | unk |
| 11 | SER10-0190 | Tomoka River | unk |
| 12 | SE15790 | Bushy Park - Back River | 0ppt |
| 13 | SE15791 | Bushy Park - Back River | 0ppt |
| 14 | SER05-389 | Waccamaw River | 0ppt |
